# Author Correction: Nonlinear shifts in infectious rust disease due to climate change

**DOI:** 10.1038/s41467-021-25692-3

**Published:** 2021-09-02

**Authors:** Joan Dudney, Claire E. Willing, Adrian J. Das, Andrew M. Latimer, Jonathan C. B. Nesmith, John J. Battles

**Affiliations:** 1 Department of Plant Sciences, UC Davis, Davis, CA USA; 2 Department of Environmental Science Policy and Management, University of California, Berkeley, Berkeley, CA USA; 3 Department of Biology, Stanford University, Stanford, CA USA; 4 U.S. Geological Survey, Western Ecological Research Center, Three Rivers, CA USA; 5 Sierra Nevada Network Inventory & Monitoring Program, Three Rivers, CA USA

**Keywords:** Climate-change ecology, Ecological epidemiology, Forest ecology

Correction to: *Nature Communications* 10.1038/s41467-021-25182-6, published online 24 August 2021.

In the original version of the published article, a phrase in Fig. 1 legend was accidentally duplicated (“(*shift in temperature denoted by change in color gradient, hot* *=* *red, cold* *=* *blue)*”). The duplicate phrase has been removed.

