# Supplementary material for: Nonlinear shifts in infectious rust disease due to climate change
Source: Nat Commun. 2021 Aug 24;12:5102. doi: 10.1038/s41467-021-25182-6 (PMC8385051; doi:10.1038/s41467-021-25182-6)
Supplement: Supplementary file 1 — Supplementary Information [file 41467_2021_25182_MOESM1_ESM.pdf]

Supplementary Information

Nonlinear shifts in infectious rust disease due to climate change

Joan Dudney*[1,2], Claire Willing[2,3], Adrian Das[4], Andrew Latimer[1], Jonathan Nesmith[5], John Battles[2]

Author information

*[1]Corresponding author, jdudney@berkeley.edu, Department of Plant Sciences, UC Davis, Davis, CA 95616

[2]Department of Environmental Science Policy and Management, University of California at Berkeley, Berkeley, CA 94720

[3]Department of Biology, Stanford University, Stanford, CA 94305

[4]U.S. Geological Survey, Western Ecological Research Center, Three Rivers, CA 93271

[5]Sierra Nevada Network Inventory & Monitoring Program, Three Rivers, CA 93271, USA

Table of Contents

Supplementary Notes
Supplementary Note 1: Blister rust life cycle and climate tolerances 3
Supplementary Note 2: Variation in R resistance genes across white pines 3
Supplementary Note 3: Drought impacts on sugar pine 4
Supplementary Note 4: Blister rust identification 5
Supplementary Note 5: Comparing quadratic and cubic models 6
Supplementary Note 6: Possible confounding of the VPD coefficient estimates 7
Supplementary Note 7: *In situ* drought study methods 7

Supplementary Tables
Supplementary Table 1. 9
Supplementary Table 2 10
Supplementary Table 3 11
Supplementary Table 4 12
Supplementary Table 5 13
Supplementary Table 6 14
Supplementary Table 7 15
Supplementary Table 8 16
Supplementary Table 9 17
Supplementary Table 10 18

Supplementary Figures
Supplementary Figure 1 19
Supplementary Figure 3 21
Supplementary Figure 4 22
Supplementary Figure 5 23
Supplementary Figure 6 24
Supplementary Figure 7 25
Supplementary Figure 8 26
Supplementary Figure 9 27

Supplementary References 27

Supplementary Note 1: Blister rust life cycle and climate tolerances

White pine blister rust is a macrocyclic heteroecious rust. It has a complex life cycle that alternates between the white pine aecial hosts and the alternate telial hosts from the genera *Ribes*, *Castellja* and *Pedicularis*[1,2]. The spores are largely wind dispersed from both hosts. Aeciospores, for example, can travel up to an estimated 1,200 km via wind currents to infect telial hosts[3] during the spring and summer. In contrast, winds carry fragile basidiospores, produced on the telial hosts, much shorter distances (< 3 km) to infect pine hosts through needle stomata during late summer[4,5].

Both temperature and moisture differently affect various parts of the complex life cycle, including the rate of infection, spore-bearing structure development, germination, and spore release. Teliospores, for instance germinate best within 4 to 9 days of age and following 12 hours of exposure to high moisture conditions at moderate temperatures (the optimum temperature range is between 10-18° C (viable range 0-22° C))[6,7]. Basidiospore production from telia begins when moisture is high (including contact with water or > 97% relative humidity), and the optimal temperature range for germination is also between 10-18° C[6,8]. Aeciospores, produced on white pine hosts in the spring, can tolerate warmer temperatures than basidiospores and teliospores ranging from 16 to 28° C[6]. Generally, blister rust reproduces best under high moisture and moderate temperatures and is therefore considered a cool weather disease[9].

Supplementary Note 2: Variation in *R* resistance genes across white pines

Here we discuss the variation in frequencies of *R* resistance genes in the four white pine hosts. We show that the proportions of resistant vs. susceptible hosts likely remained relatively stable between the surveys.

In the Sierra Nevada, frequencies of *R* resistance genes vary by species: 0.06% in sugar pine and 0.008% in western white pine[10], while whitebark pine and foxtail pines are considered the most susceptible white pine hosts[11]. The patterns of resistance are almost exactly opposite to infection rates in SEKI found following the first surveys (1995-1999): sugar pine had the highest infection rates (19.4% (#infected/#measured)), followed by western white (2.4%) and no infections were

found in foxtail and whitebark pine[12]. Resistance is even lower in alternate host understory species[2]. The low frequencies of genetic resistance suggest that selection pressures are relatively weak in the southern Sierra Nevada while host susceptibility remains high.

Assuming 33% of resistant trees died from non-blister rust causes, which is the % mortality of all uninfected sugar pines, we estimated resistance would have increased from 0.06% to 0.066% over the past nineteen years. Assuming that all resistant hosts stayed alive over the past nineteen years, which is highly unlikely, the frequency of sugar pine resistance may have increased from 0.06% to 0.098%. In addition, resistance frequencies in the recruiting population will also be relatively low for sugar pine. Only 13% of reproductive trees (>30cm DBH) died. Thus, the majority of the susceptible population was still reproducing susceptible offspring. In addition, both the length of the white pines life cycle and age-related resistance, or ontogenetic resistance[13,14] can slow selection for resistance. For example, sugar pine trees can live for over 400 years[13], and 18% of sugar pines in our plots recovered from infection[15]. Consequently, larger trees with less severe infections, as well as trees expressing ontogenetic resistance, still produced susceptible offspring[13], thereby reducing selection pressures between surveys.

While we expect that changes in sugar pine resistance were low or nominal between surveys, we did not measure genetic resistance across the population. Thus, we cannot fully eliminate the possibility that the estimated decline in blister rust prevalence due to climate change at low elevations may be a overestimate of the true climate change impact. The estimated effect of climate change at high elevations, however, is highly unlikely to be confounded by changes in resistance, as these populations likely experienced little to no selection pressures between surveys (i.e., no foxtail or whitebark pine were infected in survey plots during the first survey and only ~1% of whitebark and no foxtails were infected in the second survey).

Supplementary Note 3: Drought impacts on sugar pine

This section provides more details on the results from the *in situ* study investigating the interacting effects of blister rust and drought.

Drought occurred at least twice between surveys: first in 2007, followed by the extreme drought between 2012-2015[16] (Fig. 2c). Though our field observations of blister rust prevalence and infected host mortality did not capture the full impact of the most recent drought (2012-2015), our physiological study helped illuminate the spatially varying impacts of both the aridity gradient in SEKI, as well as the 2007 drought, that likely interacted with blister rust between surveys. Drought-induced shifts in carbon sequestration, for example, had more severe consequences for infected hosts. Infected trees had significantly less negative $\delta^{13}C$ ($F = 11.28$, $P = 0.001$) (Fig. 5a); this isotopic shift reflected a change in either the supply of CO_2, due to increasing stomatal closure in response to water deficit, and/or an increase in the demand of CO_2 in response to higher rates of CO_2 fixation[17].

Because infected individuals also had lower leaf % N ($F = 4.71$, $P = 0.03$) (Fig. 5c), stomatal closure likely drove the increase in leaf $\delta^{13}C$. Stomatal closure may have been triggered by infection sites in the main stem—hyphal growth within vascular bundles that occluded water transport[18]—resulting in increased tension in the water column. Additionally, infected trees had more negative $\delta^{15}N$ values ($F = 4.85$, $P = 0.03$) (Fig. 5b), suggesting that they experienced higher resorption of N into plant tissue, possibly a result of leaf senesce and self-pruning of infected needles/branches[19]. The combination of tighter stomatal regulation and greater changes in carbon allocation—decreasing evaporative surface area by growing significantly shorter needles and fewer fascicles (Fig. 5d,e)—indicated that infected trees experienced greater water stress at the cost of carbon acquisition[20]. The effect of infection and drought on sugar pine physiological processes may also vary depending on the location and severity of the infection. Lower crown loss in *Pinus radiata*, for example, was not correlated with carbon limitation[21], suggesting pines may have greater physiological resilience to infection when it occurs in the lower crown. Additionally, $\delta^{13}C$ has been shown to decline slightly as pine needles age[22]. Thus, while annual changes in $\delta^{13}C$ in this study appear to reflect variation in water limitation, the $\delta^{13}C$ results should be interpreted cautiously.

Supplementary Note 4: Blister rust identification

Each tree in the sample plots was evaluated for symptoms of blister rust. Crews scanned each tree searching for and counting branch and bole cankers. Branch cankers were included only if

all of the following symptoms were present: pitching, swelling or sunken bark, and discoloration of the bark on a specific section of the branch. Bole cankers were verified by the following signs: heavy pitching from a specific area, swelling or sunken bark and an “entry point” (i.e., a branch canker that clearly led to bole canker). For more details see[15].

Supplementary Note 5: Comparing quadratic and cubic models

Although our primary model specification imposes a quadratic functional form to estimate the relationship between VPD and blister rust incidence, we sought to test the robustness of these results to a more flexible functional form. To do this, we compared our estimates of the climate change effect over the past ~20 years between FE panel models with a quadratic and cubic VPD term. The outcome variable (the share of live trees in plot *i* at time *t* that were infected by blister rust) was modeled as a function of variables that changed through time, including tree density (#live trees/ha), VPD, VPD^2, VPD^3, and DBH. Specifically, the panel model had the form:

$$\ddot{y}_{it} = \beta_1\ddot{x}_{it} \;\ldots\; + \ddot{\delta}_t + \ddot{u}_{it},\; t = 1,2 \qquad (1)$$

where $\ddot{y}_i = y_{it} - \bar{y}_i$ was the time-demeaned blister rust prevalence, $\ddot{\delta}_t$ was the time fixed effect, and $\beta_1\ddot{x}_i = \beta_1 x_{it} - \beta_1\bar{x}_i \ldots$ represented the time-demeaned fixed effects. See methods section “Estimating the relationship between climate and disease prevalence” for the full model description that matched the quadratic model except for the VPD^3 term.

Whether using quadratic or cubic models, the broader interpretation of the results was similar. Though the magnitude of the climate change effect differs between models (

Supplementary Figure **2**), the direction of the climate change effect does not change. For example, when we estimated the fixed effects panel model using a cubic VPD term, we found that the direction of observed climate change effect was the same, though the magnitude of the climate change effect was, on average, greater at higher elevations and less at low elevations using the cubic model. Specifically, the quadratic model estimated that climate change increased prevalence by 6.8 (5.8-7.9) p.p. in colder regions (high elevations), while the cubic model estimated a 9.2 (8.0-10.4) p.p. Furthermore, the quadratic model estimated that climate change decreased prevalence by 5.5 (4.4-6.6) p.p. in arid regions (low elevations), while the cubic model estimated a 3.5 (2.2-4.7) p.p. However, the estimated range expansion under observed climate

change did not vary greatly between the cubic and quadratic models. The quadratic model estimated that warmer conditions over the past twenty years likely extended the prevalence into higher elevations by 777.9 (1.0-1392.9) km^2, while the cubic model estimated a 793.7 (0.96-1393.0) km^2 range expansion. Thus, the overall interpretation of our model results – that climate change led to both increases and decreases in prevalence across elevation and a range expansion of disease – does not change between the quadratic and cubic models.

Supplementary Note 6: Possible confounding of the VPD coefficient estimates

This section describes the methods used to test whether the relationship between changes in blister rust infections and changes in VPD was confounded unmeasured variables.

To test whether the relationship with blister rust infection and the independent variables changed through time, specifically, DBH, tree density, and VPD, we developed a logistic regression mixed effects model. The dependent variable was live infected and uninfected trees from the first survey and second surveys (n = 12,447), and independent variables included slope, aspect, DBH, tree density, VPD, and VPD^2. We also included species and plot as crossed random effects. All non-binary variables were standardized across data from both survey periods to have a mean of zero and a standard deviation of 1. We then interacted time (first and second survey) with DBH, tree density and VPD terms to test whether potential changes through time shifted the relationship between the explanatory variables and the outcome variable. We conducted the same analysis using a cubic model and the found the results were consistent—i.e., that the VPD coefficient estimates were not statistically different between surveys.

Supplementary Note 7: *In situ* drought study methods

This section describes in more detail the field-based needle sampling to extract stable isotopes from sugar pine and measure needle protraction in whitebark pine.

White pine host sampling:
To sample foliage from each tree, we pruned three, south-facing basal sunlight branches. We identified branch nodes and internodes to determine the needle year. Needles were extracted for

each year needles were present by counting back from the 2018 or 2017 needles along the nodes (see Supplementary Figure 9). We extracted all needles that fell within an internode. If there were any needles growing on the nodes, we did not include them in our analysis. Needles were reliably present along the internodes between the years 2012-2017 for sugar pine and between 2012-2018 for whitebark pine; only asymptomatic needles were sampled. For each needle year, we counted the number of fascicles to estimate needle retention and production. Sugar pine needles were placed in paper bags and transported to UC Berkeley where they were dried at 60°C. To estimate needle expansion, we randomly selected three dried needles from three different fascicles and measured needle length (mm). Needle expansion was estimated each year, as previous studies suggest the length does not change significantly as the needle ages[23].

Estimating change in growth across elevation:

First, we subtracted mean needle length (cm) of the drought years (2012-2015) from non-drought years (2016-2017) for both sugar pine and non-drought years 2011, 2016-2017 for whitebark pine. We then estimated the relationship between elevation and change in growth using a linear model: lm(Δ growth ~ elevation (m)). We used the function “ggpredict” to predict Δ growth across the elevation range of the observational blister rust study and extracted the range of the predicted values.

Supplementary Tables

Supplementary Table 1: Comparing model fits with different climate variables. We compared AIC values for five derived climate variables from PRISM (mean annual VPD, mean monthly VPD, precipitation, min/max temperature, and dewpoint temperature) against a null model without climate variables (no climate). We predicted first survey blister rust infection using logistic regression mixed effects models of first survey plot and tree-level data. We found that mean annual VPD had the lowest AIC value. P-values were not adjusted according to[24,25]. Please refer to our script "GLMMs_pathogen.R" for full model statistics: https://github.com/WildEcology/DudneyNatCommSEKI/tree/master/Scripts.

	**Mean annual VPD**	**Mean monthly VPD**	**Precipitation**	**Min. temps**	**Max. temps**	**Dewpoint temp.**	**No climate**
Predictors	*Coefficient*	*Coefficient*	*Coefficient*	*Coefficient*	*Coefficient*	*Coefficient*	*Coefficient*
(Intercept)	-5.81 ***	-5.60 ***	-9.51 ***	-7.67 ***	-6.52 ***	-7.87 ***	-9.67 ***
Climate variable	3.33 ***	3.36 ***	-0.77 *	1.33	3.21 ***	2.07 *	NULL
Climate variable^2	-1.06 **	-1.20 **	-0.23	-0.99 **	-0.97 *	-1.11 **	NULL
Ribes spp.	1.99 **	2.00 **	1.99 ***	1.89 ***	2.14 **	1.82 **	1.96 ***
DBH	-0.32 ***	-0.32 ***	-0.32 ***	-0.32 ***	-0.32 ***	-0.33 ***	-0.32 ***
Tree density	-0.50	-0.45	-0.67 *	-0.46	-0.58	-0.42	-0.63
Slope	-0.34	-0.36	-0.25	-0.22	-0.27	-0.18	-0.26
Aspect	-0.21	-0.16	-0.06	-0.18	-0.15	-0.10	-0.14
N	147 plot	147 plot	147 plot	147 plot	147 plot	147 plot	147 plot
	4 species	4 species	4 species	4 species	4 species	4 species	4 species
AIC	1986.9	1987.9	1996.3	1994.3	1993.0	1993.3	1998.0

* $p<0.05$ ** $p<0.01$ *** $p<0.001$

Supplementary Table 2: Fixed effects panel model results. Showing robust coefficient estimates, standard errors, and p-values (bolding = significant at $p < 0.05$) for four measured time-varying variables. Standard errors are clustered by plot and adjusted pseudo R^2 is equivalent to the GLM model pseudo R^2 with a Gaussian family[26]. P-values were not adjusted according to[24,25].

	**FE panel model with VPD**		
Predictors	*Coefficient*	*Std. Error*	*P-Value*
VPD	0.23	0.11	**0.04**
VPD^2	-0.01	0.00	**0.001**
DBH	-0.00	0.00	0.463
Density	-0.00	0.00	**0.034**
Observations	147		
Adj. pseudo R^2	0.60		

Supplementary Table 3: Logistic regression estimates, standard errors, and corresponding p-values (bolding = significant at $p < 0.05$) for GLMMs estimating first and second survey tree-level infections with plot and white pine species as crossed random effects. Pseudo R^2 values calculated using the "MuMIn" R package, which calculates a revised statistic for mixed effects models[27]. P-values were not adjusted according to[24,25].

	**First survey quadratic VPD**			**Second survey quadratic VPD**		
Predictors	*Coefficient*	*Std. Error*	*P-Value*	*Coefficient*	*Std. Error*	*P-Value*
Intercept	-5.81	0.51	**0.000**	-5.80	0.98	**0.000**
VPD	3.33	0.80	**0.000**	1.93	0.51	**0.000**
VPD^2	-1.06	0.36	**0.003**	-0.55	0.18	**0.002**
Density	-0.50	0.38	0.197	-0.05	0.21	0.809
Ribes spp.	1.99	0.66	**0.003**	0.96	0.44	**0.028**
DBH	-0.32	0.07	**0.000**	0.12	0.08	0.157
Slope	-0.34	0.28	0.223	-0.03	0.18	0.852
Aspect	-0.21	0.32	0.516	-0.48	0.21	**0.026**
N	147_{plot}<br>$4_{species}$			147_{plot}<br>$4_{species}$		
Observations	7031			5416		
Marginal R^2 / Conditional R^2	0.60/0.84			0.21 / 0.68		
AIC	1986.9			1100.5		

Supplementary Table 4: Estimated relationship between blister rust infections and maximum temperatures. Showing logistic regression estimates, standard errors, and corresponding p-values for GLMM estimating first and second survey tree-level infections with plot and white pine species as crossed random effects. Pseudo R^2 values calculated using the "MuMIn" R package. P-values were not adjusted according to[24,25].

	**First survey max. temperature**			**Second survey max. temperature**		
Predictors	*Coefficient*	*Std. Error*	*P-Value*	*Coefficient*	*Std. Error*	*P-Value*
Intercept	-6.52	0.54	**0.000**	-5.70	0.97	**0.000**
Max temperature	3.21	0.97	**0.001**	1.32	0.42	**0.002**
Max temperature^2	-0.97	0.44	**0.028**	-0.32	0.18	0.071
Density	-0.58	0.41	0.157	-0.09	0.21	0.671
Ribes spp.	2.14	0.69	**0.002**	1.05	0.43	**0.014**
DBH	-0.32	0.07	**0.000**	0.12	0.08	0.159
Slope	-0.27	0.29	0.354	-0.06	0.18	0.763
Aspect	-0.15	0.33	0.661	-0.47	0.21	**0.028**
N	147_{plot}<br>$4_{species}$			147_{plot}<br>$4_{species}$		
Observations	7031			5416		
Marginal R^2 / Conditional R^2	0.60 / 0.85			0.18 / 0.68		
AIC	1993.0			1106.8		

Supplementary Table 5: GLMM models with a cubic VPD term. Table includes coefficient estimate, standard errors, and corresponding p-values (bolding = significant at $p < 0.05$). P-values were not adjusted according to[24,25].

	First survey cubic model			Second survey cubic model		
Predictors	*Coefficient*	*Std. Error*	*P-Value*	*Coefficient*	*Std. Error*	*P-Value*
Intercept	-5.40	0.55	**0.000**	-5.86	0.98	**0.000**
VPD	3.60	1.05	**0.001**	1.93	0.50	**0.000**
VPD^2	-2.35	1.28	0.068	-0.41	0.47	0.380
VPD^3	0.45	0.38	0.235	-0.04	0.14	0.752
Density	-0.50	0.38	0.187	-0.04	0.21	0.856
Ribes spp.	2.03	0.65	**0.002**	0.94	0.44	**0.032**
DBH	-0.32	0.07	**0.000**	0.12	0.08	0.155
Slope	-0.34	0.27	0.216	-0.04	0.18	0.820
Aspect	-0.16	0.31	0.612	-0.47	0.21	**0.027**
N	147_{plot}<br>4_{species}			147_{plot}<br>4_{species}		
Observations	7031			5416		
AIC	1986.9			1102.4		

Supplementary Table 6: Fixed effects panel model results with maximum temperature. Showing coefficient estimates, standard errors, and p-values for four measured time-varying variables. Standard errors are clustered by plot and adjusted pseudo R^2 is equivalent to the GLM model pseudo R^2 with a Gaussian family[26]. P-values were not adjusted according to[24,25].

	**FE panel model with temperature**		
Predictors	*Coefficient*	*Std. Error*	*P-Value*
Max temps	0.230	0.094	**0.02**
Max temps^2	-0.009	0.002	**0.00**
DBH	-0.002	0.002	0.31
Density	0.000	0.000	0.08
Observations	147		
Adj. pseudo R^2	0.57		

Supplementary Table 7: Logistic regression estimates, standard errors, and corresponding p-values from the GLMM estimating infected trees with time (first and second survey) as interaction terms. The relationship between blister rust and VPD and VPD^2 is not significantly different between the first and second surveys (interaction terms were not significant), suggesting that the relationship between infections and climate has remained relatively stable through time. P-values were not adjusted according to[24,25].

	Blister rust model with time interactions		
Predictors	*Coefficient*	*Std. Error*	*P-Value*
Intercept	-5.61	1.06	**0.000**
VPD	2.53	0.45	**0.000**
Time	-0.17	0.30	0.574
VPD^2	-0.83	0.18	**0.000**
Ribes spp.	1.16	0.38	**0.003**
Aspect	-0.48	0.37	0.198
Slope	-0.13	0.16	0.428
Density	-0.63	0.16	**0.001**
DBH	-0.31	0.07	**0.000**
VPD*Time	-0.52	0.44	0.231
VPD^2*Time	0.23	0.17	0.169
Density*Time	1.03	0.20	**0.000**
DBH*Time	0.41	0.10	**0.000**
N_{plot}	147		
$N_{species}$	4		
Observations	12447		
Marg. R^2 / Cond. R^2	0.350 / 0.77		

Supplementary Table 8: Models explaining *Ribes* spp. occurrence. Showing regression results from two models estimating plot-level *Ribes* spp. Model 1 (left side): presence/absence (1=present, 0 = absent) of *Ribes* spp. was estimated using a logistic regression GLM with VPD, slope, and aspect as independent variables. Model 2 (right side): all variables were the same except elevation was used instead of VPD. No random effects specified because plot equaled the number of observations. Elevation was excluded in the first model because it caused unreliable coefficient estimates. Tjur's R^2 is an alternative to other pseudo-R^2 values (e.g., Nagelkerke's R^2) but used for models with binary outcomes[28]. P-values were not adjusted according to[24,25].

	***Ribes* spp. with VPD**			***Ribes* spp. with elevation**		
Predictors	*Coefficient*	*Std. Error*	*P-Value*	*Coefficient*	*Std. Error*	*P-Value*
Intercept	-0.98	0.27	**0.000**	-1.02	0.28	**0.000**
VPD	0.72	0.20	**0.000**			
Slope	0.00	0.19	0.980	0.05	0.20	0.818
Aspect	0.17	0.39	0.667	0.13	0.42	0.747
Elevation				-1.03	0.21	**0.000**
Observations	147			147		
R^2 Tjur	0.111			0.210		
AIC	171.73			157.38		

Supplementary Table 9: White pine host mortality model. Logistic regression estimates, standard errors, and corresponding p-values for GLMMs estimating the probability of infected host mortality (1=dead, 0=live) as a function of the independent variables displayed here. Includes all white pine stems (n = 448) infected at the time of the first survey. Model 1 (left side) includes VPD but not elevation, while Model 2 (right side) includes elevation but not VPD. Otherwise, the models use identical independent and dependent variables. P-values were not adjusted according to[24,25].

	**Mortality with VPD**			**Mortality with elevation**		
Predictors	*Coefficient*	*Std. Error*	*P-Value*	*Coefficient*	*Std. Error*	*P-Value*
Intercept	0.04	0.34	0.906	-0.25	0.43	0.557
DBH	-1.24	0.18	**0.000**	-1.23	0.18	**0.000**
VPD	0.70	0.27	**0.010**			
Density	0.08	0.26	0.753	0.03	0.26	0.895
Slope	0.02	0.17	0.886	0.17	0.16	0.296
Aspect	0.24	0.37	0.510	0.13	0.38	0.735
Elevation				-0.82	0.33	**0.013**
N	40_{plot} $2_{species}$			40_{plot} $2_{species}$		
Observations	448			448		
Marg. R^2/Cond. R^2	0. 25 / 0.36			0.24 / 0.34		
AIC	462.17			462.93		

Supplementary Table 10: Results from logistic GLMMs estimating second survey tree-level infections that include dead trees and dead infected trees. This model was otherwise identical (i.e., same explanatory variables) to the second survey GLMM of only live trees (Supplementary Table 3) and included plot and white pine species as crossed random effects. P-values were not adjusted according to[24,25].

	**Second survey model including dead trees**		
Predictors	*Coefficient*	*Std. Error*	*P-Value*
Intercept	-5.48	0.98	**0.000**
VPD	2.00	0.50	**0.000**
VPD^2	-0.70	0.20	**0.000**
Density	0.05	0.20	0.813
Ribes spp.	0.86	0.41	**0.037**
DBH	0.08	0.08	0.315
Slope	-0.06	0.17	0.734
Aspect	-0.52	0.20	**0.010**
ICC	0.60		
N_{plot}	147		
$N_{species}$	4		
Observations	5818		
Marginal R^2 / Conditional R^2	0.214 / 0.684		
AIC	1287.13		

Supplementary Figures

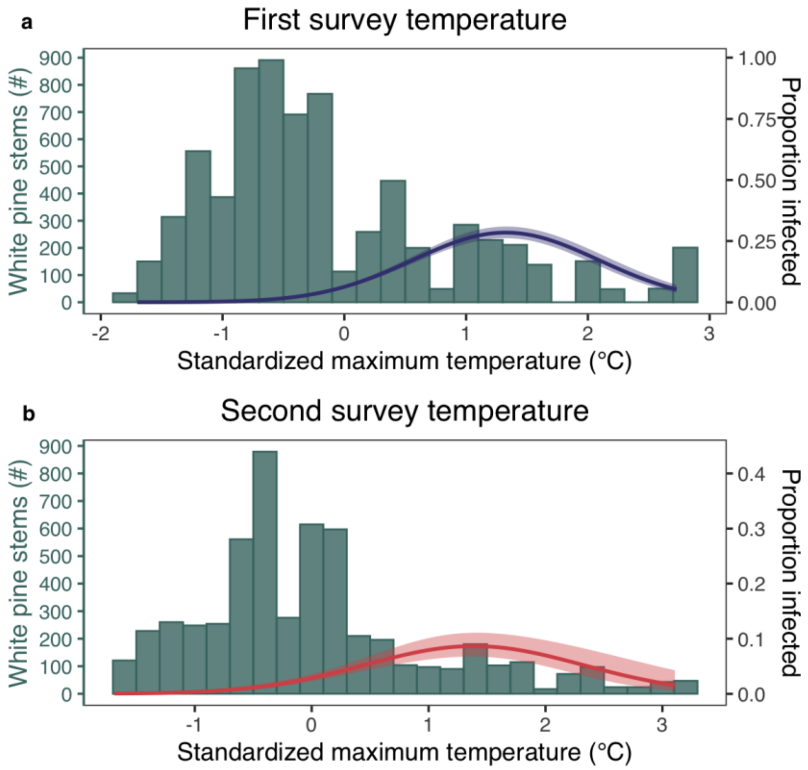

Supplementary Figure 1: Relationship between maximum temperatures and first and second survey infections. **a** Proportion of infected white pines (purple line) and the number of tree stems (green histogram bars) across standardized maximum temperature (mean of 0 with a standard deviation of 1) for the first (1995-1999) survey period. **b** Proportion of infected white pines (red line) and the number of tree stems (green histogram bars) across standardized maximum temperature for the second (2013-2017) survey period. Smoothed lines estimated using a generalized linear logistic regression with a quadratic term and shading shows the 95% C.I. band.

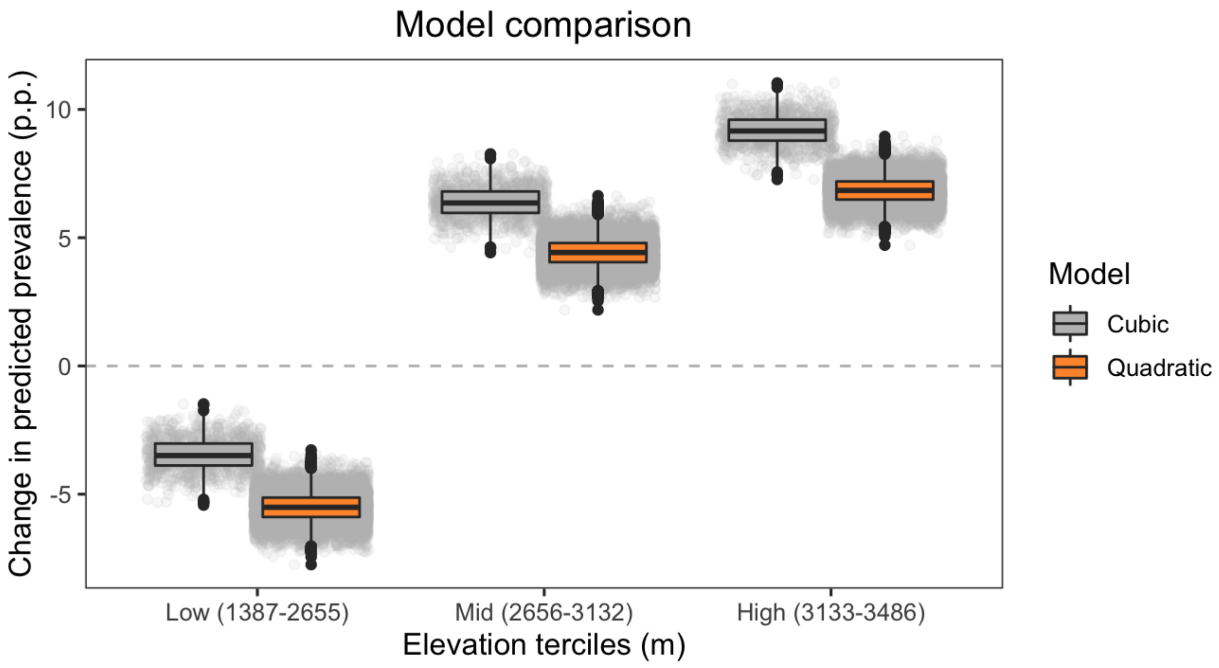

Supplementary Figure 2: Comparing the estimated climate change effect on blister rust prevalence across elevation terciles between FE panel models that included a quadratic term ("Quadratic model" grey boxplots) and cubic VPD term ("Cubic model" orange boxplots). Values are differences in predicted prevalence (percentage point (p.p.)) attributed to a change in VPD estimated by the Monte Carlo (MC) simulation for the observed climate change 2016 scenarios (n = 10,000 each boxplot). Climate change predicted prevalence was differenced from the counterfactual at the three elevation terciles (see methods section "Estimating the relationship between climate and disease prevalence"). Boxplots show the 25–75% quantile range and the 50% quantile center line. Whiskers depict data points within 1.5 times the interquartile range; includes outlier points in black and jittered data points in light grey.

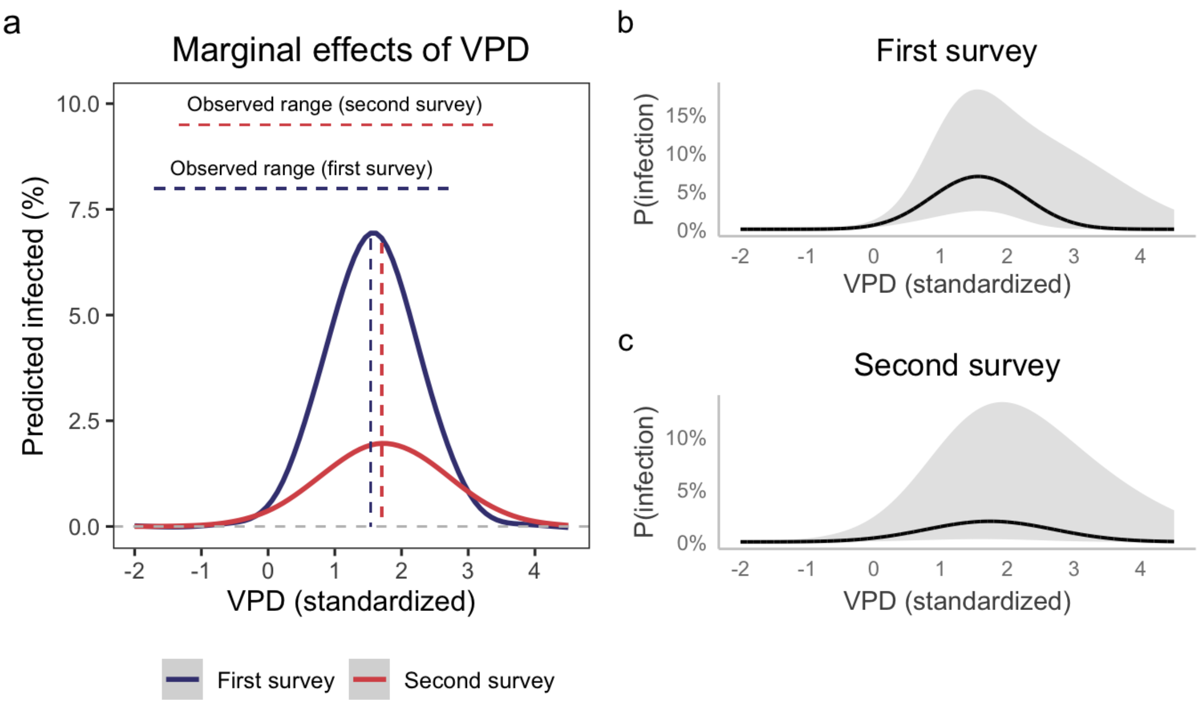

Supplementary Figure 3: Comparing the marginal effects of VPD between the first and second survey. a Displaying the predicted probability of infection using the GLMM coefficient estimates of VPD (marginal effects of VPD) for the first (blue lines) and second (red lines) surveys. Dashed vertical lines are drawn from the peak value of each line. Smoothed lines were estimated using the generalized additive mode smoothing method ("gam"). "Observed range" is the observed standardized VPD range used to estimate the blister rust-VPD relationship in the first and second surveys. **b-c** show the same predicted values displayed in panel **a** but include the 95% confidence intervals. Marginal effects calculated using the same VPD range (-2-4.5) to compare between survey periods. A slight shift to the right suggests that the climate-blister rust relationship is relatively stable and blister rust spread into higher elevations is unlikely a result of random spatial spread dynamics or local climate adaptation.

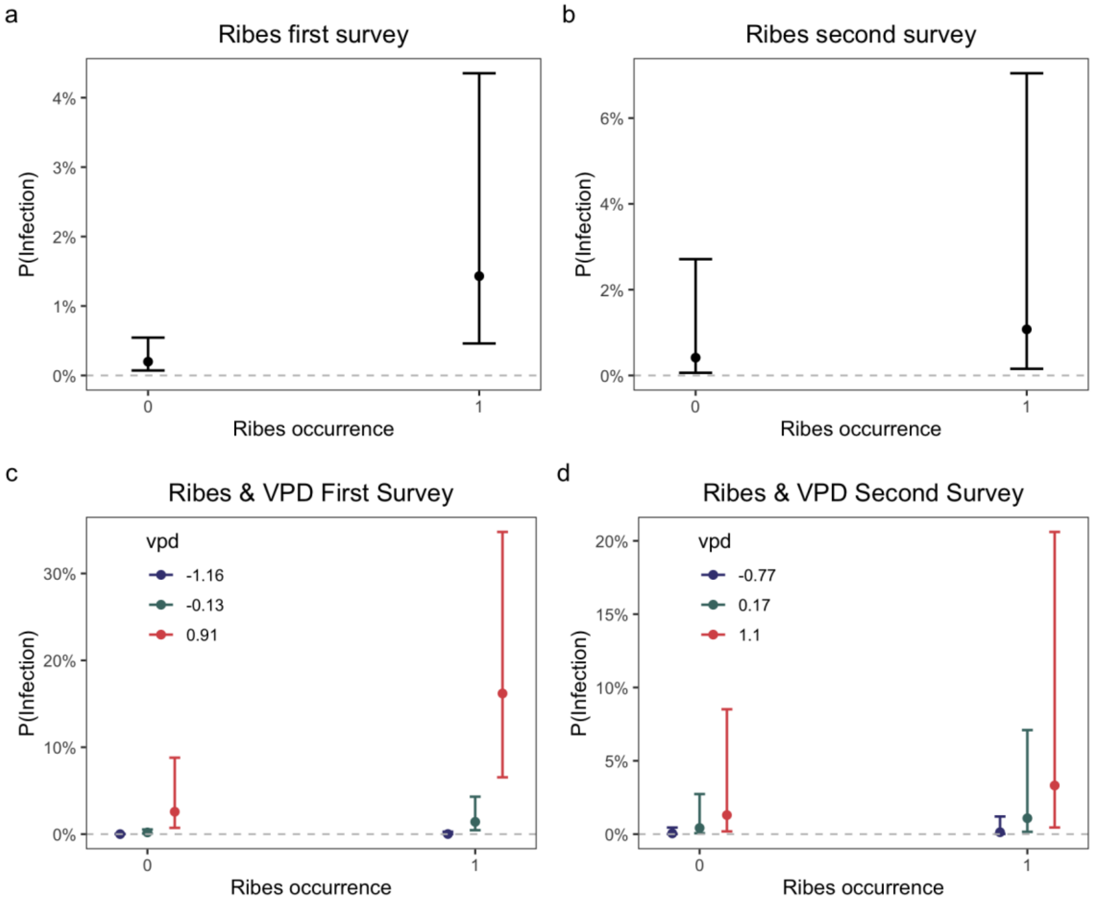

Supplementary Figure 4: Relationship between *Ribes* spp. occurrence and probability of infection estimated for the first survey (**a**) and second survey (**b**) by GLMMs of tree-level infections (Supplementary Table 3). Relationship between *Ribes* spp. and probability of infection at the VPD terciles for the first (**c**) and second survey (**d**) infections (VPD is standardized with a mean of 0 and standard deviation of 1).

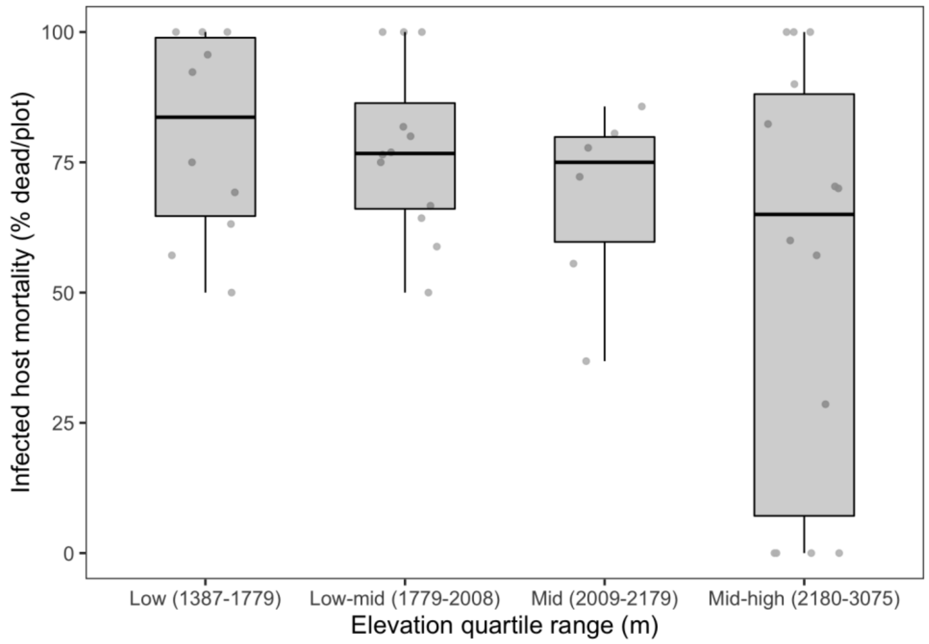

Supplementary Figure 5: Percentage of infected white pines from the first survey that died across the elevation quartile range (quartiles estimated across the elevation range where infections occurred in the first survey). Boxplots show the 25–75% quantile range and the 50% quantile center line. Whiskers depict data points within 1.5 times the interquartile range; includes jittered data points in light grey. On average, a higher percentage of total infected trees died at low elevations, 80.3% +/- 6.2 (mean ± standard error), compared to high elevations, 54.1% +/- 10.8; n = 42 plots with infected hosts at the time of the first survey.

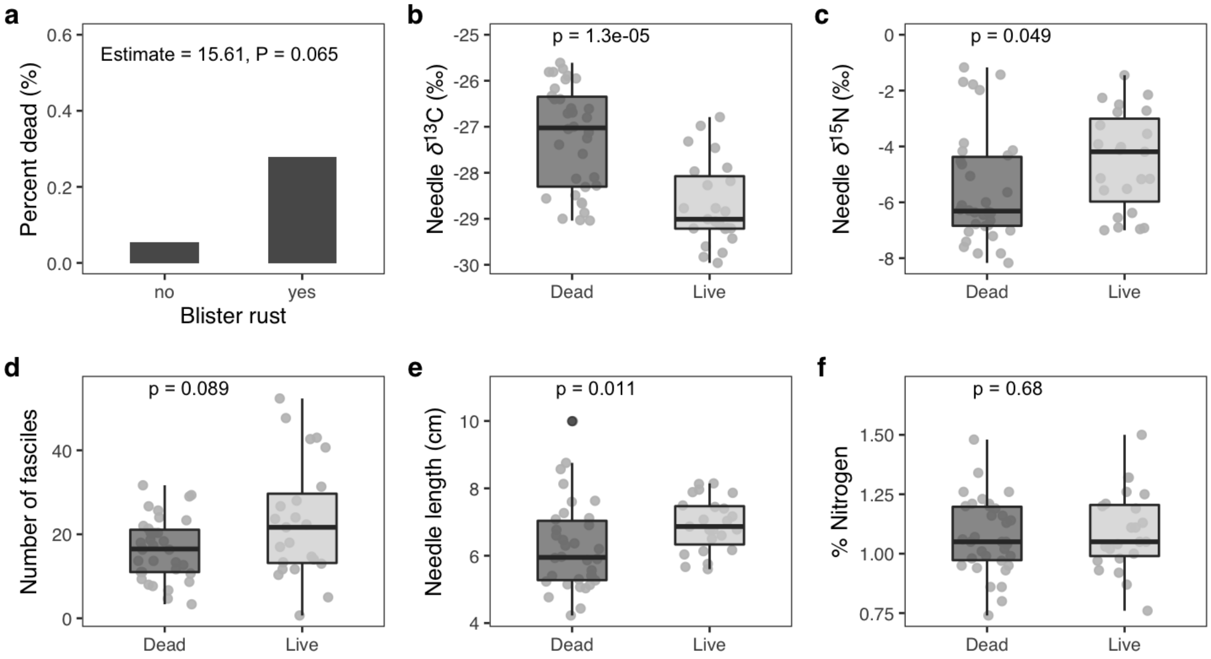

Supplementary Figure 6: Differences between infected and uninfected pairs with one or more dead tree in 2018. a The percentage of infected and uninfected trees that died (i.e., #dead/total trees). Showing coefficient estimate and p-value from logistic GLMM with blister rust infection as the explanatory variable and tree pair as the random effect (n = 36 sugar pines). **b** Boxplots depict differences between live (grey) and dead (darkgrey) paired sugar pines (n = 10 sugar pine trees). Boxplots show the 25–75% quantile range and the 50% quantile center line. Whiskers depict data points within 1.5 times the interquartile range with outlier points in dark grey. All boxplots showed jittered data points. Observations of dead trees: n = 34 needle samples. Observations of live trees: n = 23 needle samples. Specifically, boxplots show differences in $\delta^{13}C$ (**b**), $\delta^{15}N$ (**c**), the number of fascicles (**d**), mean needle protraction (**e**) and % needle nitrogen (**f**). Means statistically compared using non-parametric Wilcoxon signed rank test (two-sided).

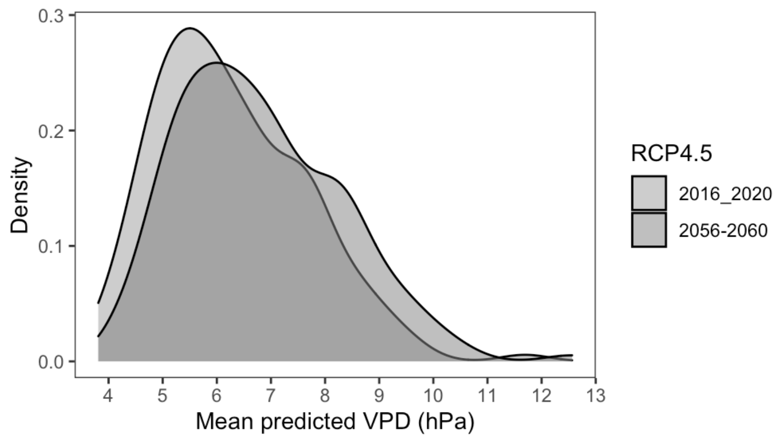

Supplementary Figure 7: Density of predicted VPD values across all plots. Showing predicted VPD (mean VPD) for each plot between 2016-2020 and 2056-2060 under RCP4.5 using the MACAv2-METDATA product GFEL-ESM2G (Geophysical Fluid Dynamics Laboratory Earth Systems Model 2) model. The MACA method downscales Global Climate Models (GCMs) to a higher spatial resolution that captures both the scales relevant for impact modelling while preserving time-scales and meteorological patterns[29].

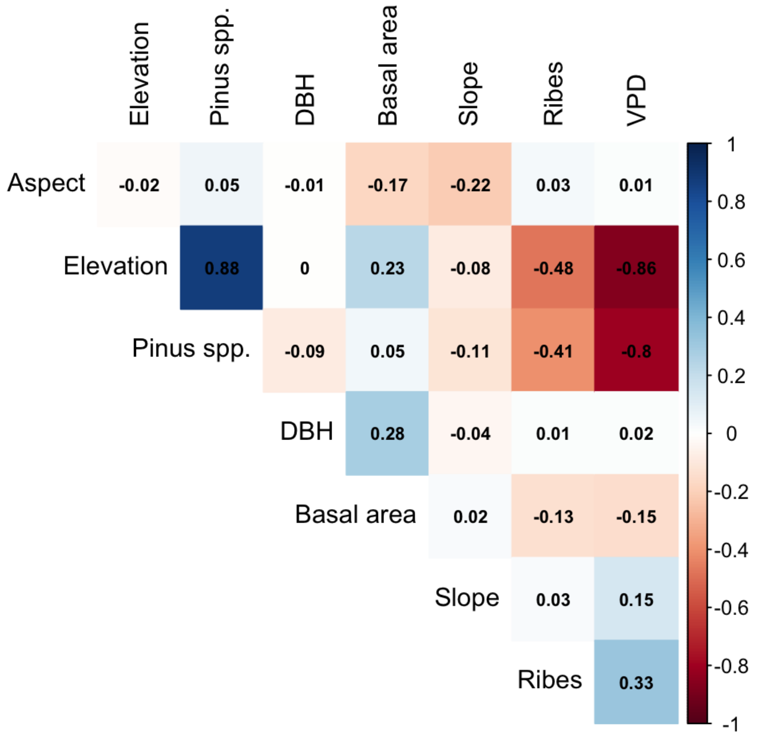

Supplementary Figure 8: Correlations among explanatory variables used in GLMM models. Showing the Pearson's correlation coefficients matrix among explanatory variables used in the GLMMs predicting blister rust infections across second survey white pines.

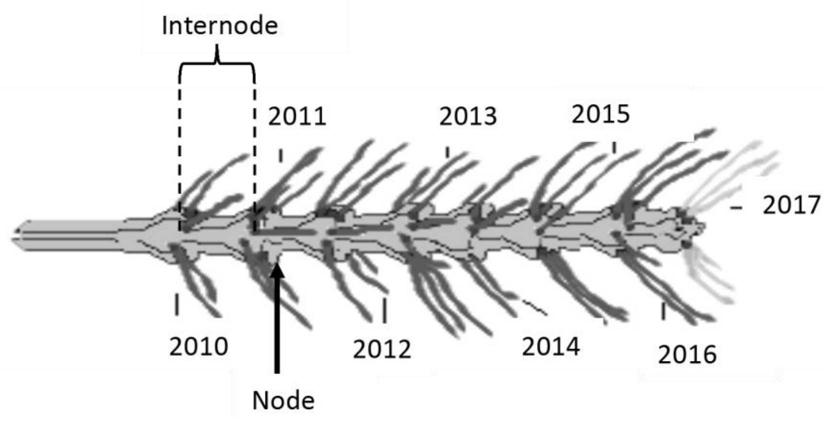

Supplementary Figure 9: Sugar pine nodes and internodes. An illustration showing how the needles from each year were identified and extracted in the field. Permission to use this figure granted by SEKI National Parks I&M.

Supplementary References

1. McDonald, G. I., Richardson, B. A., Zambino, P. J., Klopfenstein, N. B. & Kim, M.-S. Pedicularis and Castilleja are natural hosts of Cronartium ribicola in North America: a first report. *For. Pathol.* **36**, 73–82 (2006).
2. Geils, B. W., Hummer, K. E. & Hunt, R. S. White pines, Ribes, and blister rust: a review and synthesis. *For. Pathol.* **40**, 147–185 (2010).
3. Kearns, H. S. J. & Jacobi, W. R. The distribution and incidence of white pine blister rust in central and southeastern Wyoming and northern Colorado. *Can. J. For. Res.* **37**, 462–472 (2007).
4. Maloy, O. C. White pine blister rust control in North America: A case history. *Annu. Rev. Phytopathol.* **35**, 87–109 (1997).
5. Maloy, O. C. White pine blister rust. *Plant Health Prog.* **2**, 10 (2001).
6. Arsdel, E. P. V., Geils, B. W. & Zambino, P. J. Epidemiology for hazard rating of white pine blister rust. *Guyon JC Comp Proc. 53rd West. Int. For. Dis. Work Conf. 2005 Sept. 26-30 Jackson WY US Dep. Agric. For. Serv. Intermt. Reg. Ogden UT* (2006).
7. Chaloner, T. M., Gurr, S. J. & Bebber, D. P. Geometry and evolution of the ecological niche in plant-associated microbes. *Nat. Commun.* **11**, 2955 (2020).
8. Van Arsdel, E. P. Environment in relation to white pine blister rust infection. *Biol. Rust Resist. For. Trees* **1221**, 479–491 (1972).
9. Kinloch, B. B. White pine blister rust in North America: Past and prognosis. *Phytopathology* **93**, 1044–1047 (2003).
10. Kinloch, B. B. *et al.* Patterns of variation in blister rust resistance in sugar pine (Pinus lambertiana). *Schoettle Anna W Sniezko Richard Kliejunas John T Eds Proc. IUFRO Jt. Conf. Genet. Five-Needle Pines Rusts For. Trees Strobusphere 2014 June 15-20 Fort Collins CO Proc RMRS-P-76 Fort Collins CO US Dep. Agric. For. Serv. Rocky Mt. Res. Stn. P 124-128* **76**, 124–128 (2018).
11. Kinloch, B. B. & Dupper, G. E. Genetic specificity in the white pine-blister rust pathosystem. *Phytopathology* **92**, 278–280 (2002).
12. Dudney, J. Characterizing and managing drivers of change in Mediterranean forest and grassland communities. (UC Berkeley, 2019).
13. Kinloch Jr, B. B. & Scheuner, W. H. Pinus lambertiana Dougl., sugar pine. *Silv. N. Am.* **1**, 370–379 (1990).
14. King, J. N., David, A., Noshad, D. & Smith, J. A review of genetic approaches to the management of blister rust in white pines. *For. Pathol.* **40**, 292–313 (2010).
15. Dudney, J. C. *et al.* Compounding effects of white pine blister rust, mountain pine beetle, and fire threaten four white pine species. *Ecosphere* **11**, e03263 (2020).
16. Margulis, S. A. *et al.* Characterizing the extreme 2015 snowpack deficit in the Sierra Nevada (USA) and the implications for drought recovery. *Geophys. Res. Lett.* **43**, 6341–6349 (2016).
17. Farquhar, G. D. & Lloyd, J. Carbon and oxygen isotope effects in the exchange of carbon dioxide between terrestrial plants and the atmosphere. in *Stable isotopes and plant carbon-water relations* 47–70 (Elsevier, 1993).
18. Jacobs, J. J., Burnes, T. A., David, A. J. & Blanchette, R. A. Histopathology of primary needles and mortality associated with white pine blister rust in resistant and susceptible Pinus strobus. *For. Pathol.* **39**, 361–376 (2009).

19. Enta, A. *et al.* Nitrogen resorption and fractionation during leaf senescence in typical tree species in Japan. *J. For. Res.* (2019) doi:10.1007/s11676-019-01055-z.
20. Goodsman, D. W., Lusebrink, I., Landhäusser, S. M., Erbilgin, N. & Lieffers, V. J. Variation in carbon availability, defense chemistry and susceptibility to fungal invasion along the stems of mature trees. *New Phytol.* **197**, 586–594 (2013).
21. Gomez-Gallego, M., Williams, N., Leuzinger, S., Scott, P. M. & Bader, M. K.-F. No carbon limitation after lower crown loss in Pinus radiata. *Ann. Bot.* **125**, 955–967 (2020).
22. Hobbie, E. A., Gregg, J., Olszyk, D. M., Rygiewicz, P. T. & Tingey, D. T. Effects of climate change on labile and structural carbon in Douglas-fir needles as estimated by δ13C and Carea measurements. *Glob. Change Biol.* **8**, 1072–1084 (2002).
23. Ewers, F. W. Secondary Growth in Needle Leaves of Pinus Longaeva (bristlecone Pine) and Other Conifers: Quantitative Data. *Am. J. Bot.* **69**, 1552–1559 (1982).
24. Perneger, T. V. What's wrong with Bonferroni adjustments. *BMJ* **316**, 1236–1238 (1998).
25. Rothman, K. J. No Adjustments Are Needed for Multiple Comparisons. *Epidemiology* **1**, 43–46 (1990).
26. Berge, L. *fixest: Fast Fixed-Effects Estimations*. (2020).
27. Bartoń, K. *MuMIn: Multi-Model Inference*. (2020).
28. Tjur, T. Coefficients of Determination in Logistic Regression Models—A New Proposal: The Coefficient of Discrimination. *Am. Stat.* **63**, 366–372 (2009).
29. Abatzoglou, J. T. Development of gridded surface meteorological data for ecological applications and modelling. *Int. J. Climatol.* **33**, 121–131 (2013).
