# Supplementary material for: Nonlinear shifts in infectious rust disease due to climate change
Source: Nat Commun. 2021 Aug 24;12:5102. doi: 10.1038/s41467-021-25182-6 (PMC8385051; doi:10.1038/s41467-021-25182-6)
Supplement: Supplementary file 3 — Reporting Summary [file 41467_2021_25182_MOESM3_ESM.pdf]

nature research

Corresponding author(s): Dudney

Last updated by author(s): 2021/05/12

Reporting Summary

Nature Research wishes to improve the reproducibility of the work that we publish. This form provides structure for consistency and transparency in reporting. For further information on Nature Research policies, see our Editorial Policies and the Editorial Policy Checklist.

Statistics

For all statistical analyses, confirm that the following items are present in the figure legend, table legend, main text, or Methods section.

n/a	Confirmed	
☐	☒	The exact sample size (*n*) for each experimental group/condition, given as a discrete number and unit of measurement
☐	☒	A statement on whether measurements were taken from distinct samples or whether the same sample was measured repeatedly
☐	☒	The statistical test(s) used AND whether they are one- or two-sided<br>*Only common tests should be described solely by name; describe more complex techniques in the Methods section.*
☐	☒	A description of all covariates tested
☐	☒	A description of any assumptions or corrections, such as tests of normality and adjustment for multiple comparisons
☐	☒	A full description of the statistical parameters including central tendency (e.g. means) or other basic estimates (e.g. regression coefficient) AND variation (e.g. standard deviation) or associated estimates of uncertainty (e.g. confidence intervals)
☐	☒	For null hypothesis testing, the test statistic (e.g. *F*, *t*, *r*) with confidence intervals, effect sizes, degrees of freedom and *P* value noted<br>*Give P values as exact values whenever suitable.*
☒	☐	For Bayesian analysis, information on the choice of priors and Markov chain Monte Carlo settings
☐	☒	For hierarchical and complex designs, identification of the appropriate level for tests and full reporting of outcomes
☐	☒	Estimates of effect sizes (e.g. Cohen's *d*, Pearson's *r*), indicating how they were calculated

Our web collection on statistics for biologists contains articles on many of the points above.

Software and code

Policy information about availability of computer code

Data collection	No code was use to collect the field data.
Data analysis	R version 3.6.0 and QGIS 3.14. Packages and corresponding versions in R included: broom 0.5.2, clubSandwich 0.4.1, curl 3.3, egg 0.4.5, fixest 0.4.1, forcats 0.4.0, Formula 1.2-3, ggeffects 0.15.0, ggpmisc 0.3.1, ggpubr 0.2.1, ggtext 0.1.0, ggthemes 4.2.0, magrittr 1.5, margins 0.3.23, modelr 0.1.6, MuMIn 1.43.6, mvtnorm 1.0-11, nnet 7.3-12, optimx 2018-7.10, patchwork 1.0.0.9000, sandwich 2.5-1, sjlabelled 1.1.6, sjmisc 2.8.2, sjPlot 2.7.0, sjstats 0.17.5, stringr 1.4.0, tidyverse 1.3.0, zoo 1.8-6

For manuscripts utilizing custom algorithms or software that are central to the research but not yet described in published literature, software must be made available to editors and reviewers. We strongly encourage code deposition in a community repository (e.g. GitHub). See the Nature Research guidelines for submitting code & software for further information.

Data

Policy information about availability of data

All manuscripts must include a data availability statement. This statement should provide the following information, where applicable:
- Accession codes, unique identifiers, or web links for publicly available datasets
- A list of figures that have associated raw data
- A description of any restrictions on data availability

All field data for this study can be found via the Open Science Framework at https://doi.org/10.17605/OSF.IO/PC9FM110, https://github.com/WildEcology/DudneyNatCommSEKI or upon request jdudney@berkeley.edu. PRISM downscaled historic climate data can be found at https://prism.oregonstate.edu/ and MACA downscaled forecasted climate data can be found at http://www.climatologylab.org/maca.html.

Field-specific reporting

Please select the one below that is the best fit for your research. If you are not sure, read the appropriate sections before making your selection.

☐ Life sciences ☐ Behavioural & social sciences ☒ Ecological, evolutionary & environmental sciences

For a reference copy of the document with all sections, see nature.com/documents/nr-reporting-summary-flat.pdf

Ecological, evolutionary & environmental sciences study design

All studies must disclose on these points even when the disclosure is negative.

Study description

The sugar pine drought study used a paired study design of infected and uninfected trees with needle year nested within pair. The whitebark pine study used a hierarchical design with needle year nested within tree. The pathogen study used a stratified random sampling design (hierarchical) with plot and species as crossed random effects.

Research sample

The pathogen study included a stratified random sample of the four white pine populations within SEKI: western white pine, sugar pine, whitebark pine and foxtail pine. The drought study included a random sample of sugar pine and whitebark pine trees within 500 meters of the pathogen study plots and represented a subsample of the these white pine populations in SEKI. Historic climate data were downloaded from the PRISM dataset (https://prism.oregonstate.edu/) and the forecasted climate data were downloaded from http://www.climatologylab.org/maca.html. The SEKI white pine distribution map was provided by SEKI I&M program and the SEKI digital elevation model was downloaded from https://www.usgs.gov/core-science-systems/ngp/tnm-delivery.

Sampling strategy

Drought study. A total of n = 36 live sugar pine trees were identified starting within 500 meters of a randomly selected pathogen plot with observed blister rust infections. Sample size was determined from a preliminary study and informed by feasibility and cost. Budget constraints limited the study to 250 stable isotope samples, which was approximately 17 tree pairs with similar DBH (mean and standard deviation difference among pairs: 4.77+/- 4.73 cm). Sampling stopped after the 17 pairs had been measured. This was a paired study design with year nested within pair. Because whitebark pine infections were so low (~1% of the population) we only sampled live uninfected trees (n = 30).

Pathogen study: a total of 154 plots were originally established in 1995–1999 using a stratified random sampling design based on the range of white pine species within SEKI. Sample size was predicted using the formula by Cochran (1977) and informed by feasibility. Specifically, a plot containing more than 100 trees was deemed unmanageable, and even 50 trees demanded a high amount of resources. Given that trees would die over time, it was decided that a fixed sample size of 50 trees per plot be utilized, where possible, and that 30 trees would be acceptable if stand density was found to be low over a wide area. Plot size averaged 30 × 50 m, though plot length was adjusted to capture a minimum of 30 white pine stems. Plot sizes ranged from 0.1 to 3.4 ha. Crews navigated to the same plots between 2013-2017 and resurveyed each tree measuring mortality and signs and symptoms of blister rust. Sampling continued until all white pine stems and recruited individuals (>1m height) had been measured within the plot dimensions.

Data collection

Drought study: We collected 266 sugar pine and 225 whitebark pine needle samples from three low-lying, south facing, sunlit branches of each sampled tree. Needles were extracted for each year needles were present by counting back from 2017 or 2018 needles along the nodes. Sampling stopped after the 17 sugar pine pairs had been measured. All healthy fascicles were extracted along the branch within each internode, counted, placed in paper bags, and transported to UC Berkeley where they were dried at 60° C. To estimate needle expansion, we randomly selected three dried needles from three different fascicles and measured needle length (mm). 5-10 dried sugar pine fascicles were pulverized to a fine powder, weighed (8 mg), and encapsulated in tin. Samples were analyzed for both carbon (δ13C and % C) and nitrogen (δ15N and % N). Stable isotope ratios were determined for dried needle material at the University of California, Berkeley Center for Stable Isotope Ecology. All field data was recorded using pencil and paper and needle samples were collected in the field and dried and measured in the lab. Joan Dudney and Claire Willing extracted all sugar pine samples and Joan Dudney collected whitebark samples.

Pathogen study: sampling methods for the presence or absence of blister rust were adapted from Duriscoe and Duriscoe (2002), with an emphasis on consistency, so that results would be comparable across sample periods. Crews scanned all branches and the main stem of each tree from all sides searching for signs of blister rust, using binoculars on tall trees, and counted branch and bole cankers. Branch cankers were recorded if sporulating aecia or old aecial sacs were observed, or if all of the following symptoms were present: pitching, swelling or sunken bark, and discoloration of the bark on a specific section of the branch. Rodent chewing and aeciospores were included in the diagnosis when present. Bole cankers were verified by the following symptoms: heavy pitching from a specific area, swelling, or sunken bark and an entry point (i.e., a branch canker that clearly led to bole canker). While these symptoms of blister rust are not necessarily definitive, blister rust is the most probable causal agent. For crew descriptions in the first survey period, see Duriscoe and Duriscoe (2002). Sampling continued until all white pine stems and recruited individuals (>1m height) had been measured within the plot dimensions. Crews in the second survey included Joan Dudney, Jonathan Nesmith, Sean Auclair, Peter Del Zotto, Sarah Hoff-Phillips, Elizabeth Bartholomew, Vlad Kovalenko, Allyson Makuch, Matthew Mosher, Hanna Mohr, David Soderberg, Sam Zuckerman, and Matt Cahill. All data were recorded using field notes.

Timing and spatial scale

Drought study: sugar pine were sampled in summer 2017 (June-September) and resurveyed in September 2018. The area of the study was approximately 5 km2, which reflected the minimum area where ~ 18 infected sugar pines could be identified with suitable healthy pairs. The timing of this study ensured we captured consistent needle ages across sampled trees, as well as needles that spanned the drought years, 2012-2015 -- needles are not consistently retained on sunlit branches for longer than ~6-7 years on sugar pines

	Pathogen study: The first survey occurred in the summers (May-October) between 1995–1999 and the second survey occurred in the summers (May-October) between 2013-2017. Due to budget constraints, no surveys were conducted in 2014 and only approximately seven plots were sampled in 2017; ~half of the calibration surveys were conducted in 2017. The total area of the pathogen study was 3504.25 km2, which captured the range of all white pine species in SEKI. Timing of the second survey occurred ~20 years on average following the first survey and this timing reflected two critical factors: 1) feasibility and budget constraints of conducting long-term surveys in remote regions and 2) ensuring enough time had elapsed to be able to capture change in this system, which can occur at longer temporal scales due to wave year patterns and slow-growing trees.
Data exclusions	Pathogen study: Seven of the original 154 plots were excluded for the following reasons: 1) three plots were impossible to remeasure due to dramatically altered conditions and inadequate or erroneous tree location data and 2) four plots were not measured consistently through time.
Reproducibility	To ensure consistency across crews and maintain accurate field measurements throughout the season, field technicians were trained by the same forest pathologist and entomologist at the beginning of each summer in the identification of blister rust and bark beetles. In addition, a crew member from the first survey in the late 1990s trained all new crews during the second survey. Because the re-survey occurred over multiple years with different crews, approximately eight percent of the plots in the second survey were measured a second time to determine among-crew variation. On average, the difference between original and calibration surveys was a ± 0.5 canker count. This difference likely did not significantly bias our results. We did not attempt to reproduce the drought study.
Randomization	Pathogen study: plots were randomly selected within strata in GIS. Drought study: plots were randomly selected within 500 meters of pathogen study plots.
Blinding	Blinding was not relevant to this study because the data were observational of trees and disease.

Did the study involve field work? ☒ Yes ☐ No

Field work, collection and transport

Field conditions	Generally crews did not work through heavy rain or storms, so the weather conditions for the sampling and observational studies were generally warm (between 65-85°F) and moderately windy at high elevation.
Location	Drought study: Sugar pine was sampled between 36°33'10"N - 36°38'05"N and 118°45'42"W - 118°49'15"W. Whitebark pine was sampled between: 37°12'52.49"N, 118°47'23.77"W; 36°35'13 "N, 118°39'60"W; and 36°46'20"N 118°24'80"W.<br><br>Pathogen study: The study was conducted across Sequoia and Kings Canyon National Parks located in the southern Sierra Nevada, between 36°22'53.30"N, 118°45'74.74"W and 37°26'99.88"N, 118°82'89.58"W
Access & import/export	All surveys and samples were conducted following research protocols outlined by the National Parks Service and "Leave no trace" standards. All research was permitted by Sequoia and Kings Canyon National Parks Service. Drought study permit information: SEKI-2017-SCI-0029; Study#: SEKI-00469; date issued: May 30, 2017. Pathogen study permit information: SEKI-2015-2017-SCI-0045; Study#: SEKI-00469; date issued: Jun 11, 2015.
Disturbance	Branches were pruned and left hidden from human sight on the forest floor

Reporting for specific materials, systems and methods

We require information from authors about some types of materials, experimental systems and methods used in many studies. Here, indicate whether each material, system or method listed is relevant to your study. If you are not sure if a list item applies to your research, read the appropriate section before selecting a response.

Materials & experimental systems

n/a	Involved in the study
☒	☐ Antibodies
☒	☐ Eukaryotic cell lines
☒	☐ Palaeontology and archaeology
☒	☐ Animals and other organisms
☒	☐ Human research participants
☒	☐ Clinical data
☒	☐ Dual use research of concern

Methods

n/a	Involved in the study
☒	☐ ChIP-seq
☒	☐ Flow cytometry
☒	☐ MRI-based neuroimaging
